# Lithium Content and Its Nutritional Beneficence, Dietary Intake, and Impact on Human Health in Edibles from the Romanian Market

**DOI:** 10.3390/foods13040592

**Published:** 2024-02-16

**Authors:** Andreea Maria Iordache, Cezara Voica, Carmen Roba, Constantin Nechita

**Affiliations:** 1 National Research and Development Institute for Cryogenics and Isotopic Technologies—ICSI Ramnicu Valcea, 4 Uzinei Street, 240050 Ramnicu Valcea, Romania; iordache_andreeamaria@yahoo.com; 2 National Institute for Research and Development of Isotopic and Molecular Technologies, 67-103 Donat St., 400293 Cluj-Napoca, Romania; 3 Faculty of Environmental Science and Engineering, Babes-Bolyai University, 30 Fântânele Street, 400294 400535 Cluj-Napoca, Romania; carmen.roba@ubbcluj.ro; 4 National Research and Development Institute for Forestry “Marin Drăcea”—INCDS, 128 Boulvard Eroilor, 077190 Voluntari, Romania

**Keywords:** lithium, estimated daily intake, ICP-MS

## Abstract

Lithium (Li) is present in human nutrition based on food intake, and several studies recommend it for treating mood disorders, even if the biological proprieties and biochemical mechanisms represent the basis for its use as an essential element. The Li content was evaluated using the inductively coupled plasma mass spectrometry technique (ICP-MS) in 1071 food and beverage samples from the Romanian market. The results show that Li had a decreasing mean concentration in the food samples as follows: vegetables leafy > bulbous > fructose > leguminous > egg whites > root vegetables > milk products > egg yolks > meats. Approximately a quarter of all data from each dataset category was extreme values (range between the third quartile and maximum value), with only 10% below the detection limit. Mean Li concentration indicated higher values in red wine, white wines, beers, and fruit juice and lower in ciders and bottled waters. A particular interest was addressed to plants for teas and coffee seeds, which showed narrow amounts of Li. For both food and beverages, two similar matrices, including egg whites and yolks and white and red wines, were found to have significant differences, which explains the high variability of Li uptake in various matrices. For 99.65% of the analyzed samples, the estimated daily intake of Li was below the provisional subchronic and chronic reference dose (2 µg/kg_bw_/day) for adverse effects in several organs and systems. Even so, a risk occurs in consuming bulbous vegetables (Li > 13.47 mg/kg) and fructose solano vegetables (Li > 11.33 mg/kg). The present study’s findings indicate that ingesting most of the analyzed beverages and food samples could be considered safe, even if future studies regarding Li content, nutritional aspects, and human cohort diseases must be conducted.

## 1. Introduction

Lithium (Li) is naturally distributed in the environment, commonly found in various matrices, such as drinking water and food webs (especially vegetables), as input from natural and anthropogenic sources [1]. Li has valuable uses, which has determined an increasing demand on the world market that implicitly addresses environmental reasons for remediation caused by the poisoning of natural ecosystems [2,3]. Also, it has gained increased interest in renewable energy technologies, and the need to recover it from the natural ecosystems needs substantial attention [4,5,6,7]. The concentrations of Li in natural waters are related to geological substrate and human contamination due to lithium-rich brines from batteries [8]. The literature indicates a higher concentration of Li in ground waters due to washing and dissolving minerals such as clay compared with rain [9,10,11]. According to the United States Environmental Protection Agency (US EPA), Li is mentioned at the top of metals with possible risk due to insufficient reports regarding exposure in drinking water and food [12,13,14]. There is no legislation regarding the maximum Li content in edibles; even so, in Russia and Ukraine, the standard concentration is set to 30 µg/L [15]. Li was found in drinking water worldwide with a considerable variability of concentration, mainly associated with natural occurrence in geological profiles and anthropogenic contamination. Various examples can be illustrated from the United States [16], Spain [17], Lithuania [18], Austria [19], Denmark [20,21], England [22], Greece [23], North Europe [24], Argentina [25], Chile [26], Japan [27,28], and Nigeria [29]. Also, in plants, the Li content is variable, and physiological studies indicate that low concentrations stimulate growth, compared with high concentrations that induce toxicity in various doses depending on species, individuals, or organs [30]. Most plants accumulate significant Li content in tissues and tolerate high daily intake doses [31]. The reports show that grains and vegetables assimilate high Li concentrations up to 4.6 µg/g [32,33]. Compared with low content in onions, green chilies, cauliflower, rice, and wheat, high Li was found among coriander leaves, tomatoes, garlic, nutmeg, and cumin seeds [34]. The concentration in tea plants was between 0.1 and 0.4 mg/kg [35]. Some studies present the concentrations of Li in mushrooms, ranging up to 0.189 mg/kg, which is not high considering these fruit bodies are extremely capable of monitoring and remedying polluted ecosystems and assimilating toxic and potentially toxic elements [36,37].

Nutritional studies suggest the beneficial role of Li as an essential trace element for human dietary intake [18,38]. Li-induced toxicity mechanism in humans is very complex, with no significant scientific results known, even if an extensive interest exists due to its considerable use in emerging industries. The provisional daily dose for humans is recommended not to exceed 1 mg/day [32]. Some studies discuss the positive effects of Li assimilated naturally from drinking water, which may increase human lifespan [39]. Although natural Li ingestion is significantly lower than therapeutic ones, it was documented that low content in drinking water consumed daily is correlated with an anti-suicidal effect [40,41]. Li is an antiepileptic antipsychotic commonly used as a preventive in bipolar disorders and unipolar depression [42,43,44]. The Li mechanism in the brain is still unclear, even if it is known that it acts beneficially on mood disorder episodes and suicidal behavior [45]. Even more, it was noted that Li in high amounts ingested periodically can be associated with lower rates of mortality in patients diagnosed with affective disorders [46,47]. Other reports indicate that long-term treatment with Li increases exposure, which induces toxicity [48]. Thus, not only the positive effects are reported in the literature, since experimental data correlate Li used for therapy with function disruption in vital organs, including the heart, kidneys, thyroid gland, reproductive and endocrine systems, or renal function [49,50,51,52]. The oral intake of Li toxicity can vary from mild to chronic, and the tolerance level differs among individuals [53]. Based on the high Li toxicity, the recommendation for therapeutic dose is usually between 5.6 and 8.4 mg/L, and the toxicity doses overhead the concentration of 10.5 mg/L [54].

Agricultural and industrial practices, including other anthropogenic activities such as waste deposits, are responsible for extensive pollution in the environment, including with Li. This alkali element is retained in the food web and possibly interacts negatively with human health in high amounts, so multiple studies are required to report and monitor concentration worldwide. The present study aimed to investigate the Li content in food and beverages from the Romanian market and assess the estimated daily intake index (EDI) reported to provisional reference doses (p-RfD) that screen chronic and subchronic exposure for humans associated with Li ingestion. A comparison between Romanian findings and the Li level from similar matrices around the globe was computed as a reference for the database created.

## 2. Materials and Methods

### 2.1. Sampling and Preparation

Drinks, food, and vegetables were acquired from Romanian supermarkets and included in the analysis during 2021–2023. The matrices investigated were chosen considering the food commonly found in basic daily dietary programs. Thus, we analyzed Li content in 752 food samples, including meat (*n* = 130), egg white (*n* = 120), egg yolk (*n* = 120), milk products (*n* = 80), bulbous vegetables (*n* = 40), root vegetables (*n* = 140), fructose solano vegetables (*n* = 60), leafy vegetables (*n* = 34), and leguminous vegetables (*n* = 28). Other 319 samples included red wines (*n* = 32), white wines (*n* = 80), beers (*n* = 52), fruit juices (*n* = 35), ciders (*n* = 32), bottled waters (*n* = 15), plants for teas (35), and coffee seeds (*n* = 38). Amounts of 250–500 g ground dry coffee and tea were used in the analysis. Ultrapure deionized water (18 MΩ cm^−1^) from a Milli-Q analytical reagent-grade water purification system (Millipore) and ultrapure nitric acid (Merck KGaA, Supelco, Sigma-Aldrich, Darmstadt, Germany, 60%) were used. All plastic containers, pipettes, and reagents were new or cleaned by soaking them for 24 h in nitric acid HNO_3,_ *w* = 10%, p.a grade, Merck KGaA, Sigma-Aldrich, Supelco, Darmstadt, Germany, and washing them with ultrapure water. A solution with Mg, Cu, Rh, Cd, In, Ba, Ce, Pb, and U (10 µg/L) from Perkin-Elmer (Pure Plus, Billerica, Massachusetts, USA), for optimization procedures and a high-purity inductively coupled plasma (ICP) multielement standard solution (included Li) obtained from Perkin Elmer Life and Analytical Sciences (Billerica, Massachusetts, USA) for the calibration curve in the quantitative analysis were used. Each sample was analyzed in duplicate, consisting of 10 replicates. Since samples have a very complex composition, the total digestion of the matrix is mandatory to ensure complete metal solubility. Transformation of solid pieces into homogenous liquid phase before sample analysis is first required. Before elemental analysis, the food samples were oven-dried at 60 °C for 12 h until a constant weight was obtained. Then, the dried samples were ground in a stainless-steel mill until fine particles that could pass through a 0.45 mm mesh were obtained. The internal laboratory method for digestion of samples was optimized to be 0.1 g of sample and 3 mL of 65% nitric acid HNO_3_, p.a grade. A microwave oven model Speedwave (Berghof Products and Instruments Ltd., Eningen, Germany) was used to digest food samples for quantitative concentration. The digestion was performed with the controlled program (pressure and temperature) for 12 min at 200 °C. After complete digestion and cooling, the samples were filtered, transferred to 50 mL graduated polypropylene tubes, and diluted to volume with deionized water.

The trueness of the recovery and precision as the relative standard deviation of the procedure were determined by analysis of the certified reference materials. The selected CRMs cover matrices with Li contents as follows: NCS DC73349-Trace elements in bush branches and leaves (NIM-GBW07603), National Analysis Center for Iron & Steel NACIS and NCS ZC73031 Carrot—NACIS, Beijing, China. Li is certified in NCS DC73349-Trace elements in bush branches and leaves with a mass fraction of 2.60% and in NCS ZC73031 Carrot with a mass fraction of 0.16 ± 0.02%. About 0.2000 g of CRM were weighed into PTFE digestion vessels for each replicate. After adding chemicals, the microwave program was set to a maximum temperature of 200 °C, reached within a ramp time of 12 min, and then held for 15 min. The recovery was higher than 85%, which testified to the applicability of the method to our edibles. The relative standard deviations were less than 10% for Li determined. The limit of detection (LOD) and limits of quantification (LOQ) were calculated by three and ten times the standard deviation of the blank sample divided by the slope of the analytical curve, respectively. The performance parameters were LOD—0.001 mg/kg and a relative standard deviation of less than 0.5%.

The performance parameters (sensitivity, linearity, precision, accuracy, and recovery) of the method of element determination were presented by Voica et al. [55]; linearity was established using a calibration curve, R > 0.9999; limit of quantification (LOQ): 10.9 ng/L. The accuracy as the recovery and the precision as the relative standard deviation of the analytical technique were evaluated by analyzing a certified common reference material (ICP-multielement standard solution XXI, concentration 10 mg/L, 30 element in HNO_3_ suprapur 6 %, Merck, KGaA, Darmstadt, Germany). The mean relative standard deviations of replicate samples were under 10%. The agreement between the certified and measured values was good, demonstrating the satisfactory performance of the developed method. An inductively coupled plasma-mass spectrometer (ICP-MS, Perkin Elmer ELAN DRC e, Waltham, MA, USA) instrument was used with a Meinhard nebulizer and glass cyclonic spray chamber for pneumatic nebulization, and the gaseous argon used to form the plasma was of 4.8 purity. The operational conditions were optimized using a tuning solution (Elan 6100 Setup/Stab/Masscal Solution 10 μg/L Ba, Cd, Ce, Cu, In, Mg, Pb, Rh, U, from Perkin Elmer). Each sample was analyzed in duplicate, consisting of ten replicates. The operating conditions were nebulizer gas flow rate, 0.92 L/min; auxiliary gas flow, 1.2 L/min; plasma gas flow, 15 L/min; lens voltage, 7.25 V; radio frequency power, 1100 W; CeO/Ce, 0.030; and Ba++/Ba = 0.28.

### 2.2. Health Risk Assessment

The daily intake (mg/day) of Li was estimated by multiplying the Li concentration in beverages (mg/L) and fresh food samples (mg/kg fw) and the daily ingestion rate of beverage and food (L/day or kg fw/day) [56,57]. The estimated daily intake (µg/kg_bw_/day) of Li was calculated by using the following Equation (1) [56,57].

(1) $$EDI=\frac{C\cdot IR}{BW}$$

where *C* is the Li concentration in beverages (µg/L) and fresh food samples (µg/kg fw), *IR* is the daily ingestion rate of beverage and food (L/day or kg fw/day), and *BW* is the average body weight of an adult person (70 kg) [57]. The daily ingestion rate was calculated based on the data on annual food consumption at the national level published in the National Institute of Statistics reports, namely: 114.5 L/year for bottled water, 88.1 L/year for beer, 23.7 L/year for wine, 115.1 L/year for refreshing drinks, 2.3 kg/year for coffee, 37.8 kg/year for meat; 243 pieces/year for eggs, 15.4 kg/year for root vegetables, 45.1 kg/year of fructose solano vegetables, 22.3 kg/year of bulbous vegetables, 19.4 kg/year for leguminous vegetables, 19.3 kg/year for leafy vegetables, and 15.8 kg/year for fruit [58,59].

The *R_f_D* is a measure with a recognized uncertainty that evaluates the value of a daily dose capable of producing health effects during a human lifetime. The *R_f_D* evaluates the most significant and sensitive lowest observed adverse effect level (*LOAEL*) for noncancer effect and is corrected by uncertainty and modifying factors (Equation (2)).

(2) $$R_{f}D=\frac{LOAEL}{{UF}_{A}\times {UF}_{H}\times {UF}_{L}\times {UF}_{S}\times {UF}_{D}\times MF}$$

where *UF_A_* is an incertitude associated with using experimental findings obtained by in vivo animal tests in evaluating human exposure; *UF_H_* is a factor that quantifies multiple variables capable of inducing high variability in human resistance to stressors, including age, gender, genetic adaptation, body weight; *UF_L_* is the expected ratio of *LOAEL*; *UF_S_* the incertitude associated with estimating chronic exposure based on subchronic exposure; *UF_D_* represents the incertitude determined by the probability of association with an unequal dataset; and *MF* is a modifier factor which raises the incertitude limits by adding supplementary buffer explained by not included possible unknown aspects which can add adverse human effects not taken into account by the standard uncertainty factors previously described. The present case study calculated the provisional subchronic and chronic *RfD* for Li based on the *LOAEL* reference for adverse effects in several organs and systems divided by an uncertainty of 1000. The value of 2.1 mg/kg-day serum Li ingestion has been established by provisional peer-reviewed toxicology values (PPRTVs) as a base for derivation since 2008.

## 3. Results and Discussions

### 3.1. Analysis of Li Concentration in Food and Beverages

**Figure 1:**
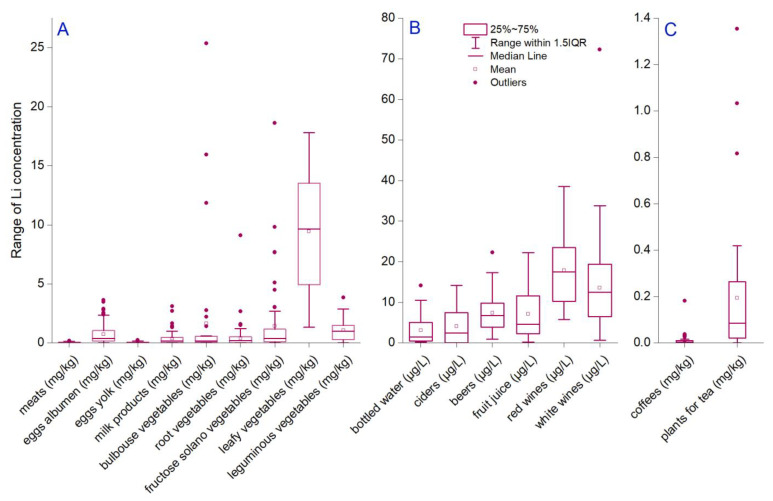
Statistical distribution of Li concentration in (**A**) food samples (meats, egg whites, egg yolks, milk products, bulbous vegetables, root vegetables, fructose solano vegetables, leafy vegetables, and leguminous vegetables); (**B**) beverages (bottled water, ciders, beers, fruit juices, red wines, and white wines); (**C**) seeds and plants for beverages (coffees and teas).

The major constituents of food are classified into seven groups: proteins, carbohydrates, fats, minerals, vitamins, fiber, and water. Li has not been considered an essential trace element; even more, there is a gap in knowledge regarding biological importance and molecular behavior [32]. Even so, it has been demonstrated that low concentrations are associated positively with biological activities in plants, animals, and humans [39,60]. On the contrary, a high dose of Li correlated with toxicity and side effects [61,62]. Various studies indicated a human Li dietary intake of 1000 µg/day [63,64]. Considering this recommendation, our study analyzed different food and beverage matrices usually consumed to understand whether or not this recommendation can be achieved. The results show that mean Li content decreased in vegetables leafy > bulbous > fructose > leguminous > egg white > root vegetables > milk products > egg yolk > meats (9.43 ± 4.92; 1.64 ± 4.92; 1.42 ± 3.01; 1.08 ± 1.08; 0.72 ± 0.80; 0.40 ± 0.84; 0.35 ± 0.55; 0.03 ± 0.05; and 0.03 ± 0.03 mg/kg) (Figure 1A). A further assessment of possible amounts of Li daily intake reveals that a higher dose can be reached through the values assimilated with the third quartile (q3). The q3 had a decreased order in the above-enumerated matrices, which were 13.52, 0.56, 1.16, 1.47, 1.03, 0.51, 0.44, 0.06, and 0.05 mg/kg. Also, it was observed that approximately a quarter of all data from each matrix was higher than the third quartile up to maximum values (extreme values). Thus, based on extreme concentrations assimilated through food ingestion from the Romanian market, it is not likely to contain excessive Li. Even more, one can compare the extreme values with those only 10% below the detection limit. As a maximum reference limit, we stated that the highest values were found in bulbous vegetables, fructose solano vegetables, leafy vegetables, and root vegetables, ranging up to 25.35, 18.62, 17.80, and 9.09 mg/kg, which are daily used in at least one main dish.

Li content in two matrices with a high degree of similarity, egg white and yolk, was subjected to a one-way ANOVA test for mean comparison, and significant differences were found (*F*-value = 88.38; *p* < 0.001). Also, Levene’s test for homogeneity of variance check indicated a significant difference (*F*-value = 164.75; *p* < 0.001). This result can be beneficial for the cases of consuming only parts of eggs, which is a widespread habit. The demand for egg white products is very high since they contain a significant protein content and are less allergenic than egg yolk [65,66]. Thus, the literature indicates a price up to 8 times higher for egg whites based on the increased demand for fat- and cholesterol-free high-quality row protein [67,68]. Li content in egg whites and yolks was previously analyzed for Romanian products, and mean concentrations of 0.31 and 0.11 mg/kg are comparable to our results [69]. High Li contents in eggs were observed in the literature, e.g., as average in Spain (2.48 ± 0.74 mg/kg) [17] and Italy (0.003 mg/kg) [57], respectively, with maximum value in Germany (6.50 mg/kg) [70]. The concentration in those regions is explained through Li deposit belts, which are further transferred from the environment through soil and water to the food chain [71,72,73].

A synthesis of the literature to determine the interval of variation, geographic variability, and even food inter-categories diversity shows a high Li content in Spain meat, as is the case of ham (4.42 ± 0.78 mg/kg), chicken drumsticks (2.13 ± 0.56 mg/kg), chicken breast (2.58 ± 1.42 mg/kg), rabbit (1.89 ± 0.56 mg/kg), pork (3.44 ± 1.93 mg/kg), and beef (2.61 ± 0.55 mg/kg) [17,74]. Quantitative Li variability is associated with geological chemical composition, human contamination, and organ bioavailability and storage [72,75]. The multiple sources (e.g., water, grains, vegetables, grass, salt) of assimilating alkali metals in meat can be assumed. In comparison, lower values were reported for pork meat in Romania (0.002 ± 0.001 v) [55], Republic of Korea (0.05 ± 0.01 mg/kg) [76], and Italy (0.002 mg/kg) [57]. For milk and dairy products, the concentrations were 2.08 ± 1.26 mg/kg (whole yogurt), 2.42 ± 1.53 mg/kg (skimmed yogurt), 0.87 ± 0.03 mg/kg (whole milk) [17], 0.002 mg/kg (milk and yogurt) [57], 0.07 ±0.04 mg/kg [77], and 3.62 mg/kg [70]. A difference between bovine (0.31 ± 0.02 mg/L) and ovine (1.88 ± 0.13 mg/L) milk was emphasized in Romanian studies [55]. The milk-based cheeses analyzed in the literature show the highest Li values for France’s fresh products (4.02 ± 1.94 mg/kg) and Spain’s hard cheeses (1.53 ± 0.49 mg/kg). Fresh and aged cheese in Italy shows no differences (0.008 and 0.004 mg/kg) [57], as well as bovine and ovine cheese (0.08 ± 0.007 and 0.57 ± 0.04 mg/kg) [55].

Analyzing the vegetables, we observed high amounts in peas (4.17 ± 0.45 mg/kg), beans (2.66 ± 1.10 mg/kg), tomatoes (2.88 ± 0.95 mg/kg), chickpeas, lentils (1.89 ± 1.19 mg/kg), and black and white potatoes (1.53 ± 0.73 and 0.74 ± 0.38 mg/kg) [17] from Spain, and Poland (2.30 mg/kg) [64]. In Italy, the amount of Li was low for tomatoes (0.002), potatoes (0.008 mg/kg), onions and garlic (0.002 mg/kg), and cabbage (0.01 mg/kg) [57]. The mushrooms are very sensitive, also known as bioindicators of environmental pollution, and assimilated Li at 0.19 mg/kg in Poland [64], 0.11 ± 0.06 in Hungary [36], and 0.008 in Italy [57]. Fruits represented by apples and bananas had 1.33 ± 1.09 and 0.95 ± 0.27 mg/kg [57]. A very high Li content in fruits was noted in Germany, 6.70 mg/kg [70]. Caramel creme in the dairy desserts had a Li content equivalent to 1.29 ± 0.57 mg/kg. Cereals such as bread had values of 5.42 ± 0.92 mg/kg [17] and 0.01 mg/kg [57]; bread and cakes of 0.61 mg/kg [70]; breakfast cereals 3.15 ± 2.17 mg/kg [17], 0.88 mg/kg [70], 4.4 mg/kg [77]; pasta and grain 0.01 mg/kg [57]. The almonds and peanuts had 10.46 ± 6.69 and 8.95 ± 6.46 mg/kg in the USA, Spain, and Turkey [17], the soybean grains and seeds in Brazil contained between 8.00 and 11.20 mg/kg and in Poland had 8.80 mg/kg [78]. The sweet cakes had a significant Li content of 2.16 ± 0.90 mg/kg in Spain [17], compared with chocolate, candy bars, cakes, pies, and pastries (0.006 mg/kg), and biscuits and dry cakes (0.008 mg/kg). The honey contained maximum values of 0.68 ± 0.07 mg/kg [79] in Turkey, 4.26 mg/kg [80] in Saudi Arabia, and 1.20 mg/kg [67] in Slovenia. The fats and oils had values from below instrumental limits [17] to 0.006 ± 0.0005 in Romania [55], 0.21 × 10^−3^ in olive oil, 0.87 × 10^−3^ in vegetable oils, and 0.002 in butter measured in samples collected from Italy [57]. The examples presented show that uptake and sensitivity to Li are species-dependent, with high levels in vegetables and animals connected through a food web chain. A geographical distinction can be observed since extreme amounts of Li are comparable in various products with similar origins.

### 3.2. Lithium in Beverages

The concentration of metals, including Li, in alcoholic beverages can significantly affect their consumption, derived from the negative to positive effects caused by their presence. Thus, our study analyzed mean Li concentration in 319 beverage samples, resulting in higher values in red wines (17.88 ± 8.70 µg/L), white wines (13.55 ± 10.33 µg/L), beers (7.38 ± 4.76 µg/L), and fruit juices (7.11 ± 6.27 µg/L) (Figure 1B). Lower values were measured in ciders (4.05 ± 4.83 µg/L) and bottled waters (3.10 ± 4.08 µg/L). A particular category was designated for plants for teas (0.19 ± 5.72 mg/kg) and coffee seeds (0.01 ± 0.03 mg/kg) (Figure 1C). The one-way ANOVA analysis indicated significant mean differences, even if variances are not significantly different between similar beverages, i.e., red and white wines. The third quartile indicates a decreasing order of beverages from red wines > white wines > fruit juices > beers > ciders > bottled waters (23.52 > 19.36 > 11.59 > 9.82 > 7.47 > 5.06 µg/L). A low Li content was found based on q3 statistics in tea plants (0.26 mg/kg) and coffee seeds (0.01 mg/kg). The maximum Li concentrations in increasing order were as follows: 0.18 mg/kg (coffees), 0.26 mg/kg (plants for tea), 14.08 µg/L (bottled waters), 14.18 µg/L (ciders), 22.26 µg/L (fruit juices), 22.28 µg/L (beers), 38.54 µg/L (red wines), and 72.26 µg/L (white wines). The percentage of extreme values (between q3 and maximum) was higher for coffees (34.21%), plants for tea (27.77%), and bottled water (26.66%), respectively; the lowest were calculated for ciders (18.18%).

Bottled water is essential for life in industrialized countries; there is an increasing trend in its consumption [81]. Fifteen bottled natural mineral waters purchased from supermarkets by different producers were characterized in this study. The mean Li concentration of 3.10 µg/L, ranging between 0.185 and 14.09 µg/L, was close to other reported values [17,70]. The results found in the literature show that mineral waters represent an essential source of Li [82], contributing significantly to Li intake, approximately 35% for adults. Coffee and hot beverages are associated with 17% and 14% of the total Li assimilated by ingestion [83]. In the European Union, Li in potable water has no current regulations, but future recommendations are expected in the coming years. In Australia, it is regulated as a pollutant limited to 2.5 mg/L for water used in irrigation [84]. On the other hand, Li in drinking water, even if it is not recognized as an essential trace element, is limited to 1000 μg/day as a reference for 70 kg body weight. In dietary practice, this recommendation is difficult to apply. Limited data exist on how frequently consumed beverages contribute to Li intake in the human body. In contrast, there are multiple studies related to Li concentrations in public drinking water (tap water) in, respectively, Greece (0.1–121 µg/L) [23], Austria (<3–1300 µg/L) [85], Lithuania (0.5–35 µg/L) [18], Italy (0.11–60.8 µg/L) [86], Denmark (0.6–30.7 µg/L) [21], Portugal (< 1–191 µg/L) [87], and in bottled waters which are richer in Li, for example, 9.86 mg/L (Slovakia) and 5.45 mg/L (Armenia) [15].

The variation interval of Li concentration in studied beers was 0.96–22.28 µg/L, with a mean of 7.38 µg/L. The low values are explained by the water content used in the beer production process, which is demineralized and loses trace elements, including Li [81]. The mean analyzed Li concentration of 14.79 µg/L in wine (red wine: 17.88 µg/L; white wine: 13.55 µg /L) was insignificantly higher than values of 11.6 µg/L reported in German wines [88], or 10.04 and 3.1 µg/L in red and white wines in Italian regions [57]. Three of the analyzed wine samples revealed high Li content (72.26 µg/L for a red wine and 38.14 and 32.36 µg/L for two white wines), possibly associated with soil geochemistry. Considering the high value of the metals related to vegetable products in human nutrition, various studies analyzed the composition and processes, mainly to quantify human nutrition’s safety and quality [89,90]. Herbs for tea are applied in folk homeopathic medicine to treat various diseases [91]. Multiple health and therapeutic activities are associated with consuming vegetables, so evaluating elemental content represents a fundamental activity of determining the quality for use in daily diet [91,92]. Also, coffee is a significant source of essential trace elements, including non-essential and toxic metals, consumed by humans. Determining coffee’s contamination status can help evaluate its possible effects that may affect human health [93].

Tea and coffee are commonly consumed beverages, and several studies consider that medicinal teas may enhance the human Li supply [94]. Also, it is necessary to evaluate Li bio-accessibilities from edibles [95], such as herbal samples used as tea belongings and distinct pure-origin roasted and ground coffee samples. In these matrices, the concentration varied from <0.001 to 1.35 mg/kg for herbal samples and <0.001 to 0.18 mg/kg for coffee samples, with a mean value of 0.19 and 0.01 mg/kg, respectively, comparable with similar results [35,71]. Different interpretations of results can be associated with vegetables such as coffee and tea that are typically prepared with water, which can cumulate the total amount of Li from two sources. Since the beginning of the 20th century, lithiated drinks have been on the market and considered beneficial for human health based on their high Li content [81]. In 1929, “Lithiated Lemon Soda” was a refreshing drink supplemented with Li (5 mg of Li citrate). Still, in 1948, it was banned and considered toxic [71]. These drinks are still on the market but contain only 1.4 µg/L of Li. Our study presents results for fruit juices, with a Li concentration varying from 0.16 to 22.26 µg/L, with a mean of 7.119 µg/L. The literature shows data regarding Li content in soft drinks; for example, most cola-based drinks have low content, except one (Afri Cola) with over 50 µg/L, or energy drinks contain an average of 20.90 ± 37.3 µg/L. An extreme value was found as an exception for one with acai extract that has over 100 µg/L [71,96]. The present study could help evaluate Li intake via beverages in the Romanian population and represent a national fingerprint reported to regional differences [97].

### 3.3. Estimated Daily Intake (EDI) of Li via Food and Beverage Ingestion

**Figure 2:**
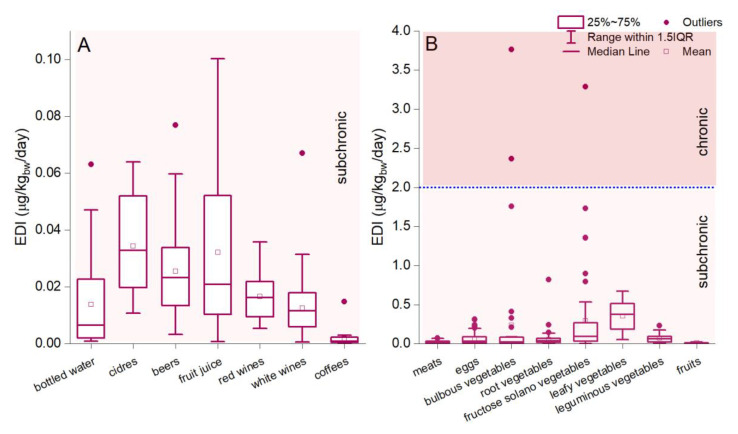
Estimated daily intake (µg/kg_bw_/day) of Li via ingestion of food (**A**) and beverages (**B**). The blue dotted lines represent the subchronic and chronic p-R_f_D.

The health risk assessment represents an essential tool for understanding health risks in human activities and indicating evidence for decision-makers. The EDI evaluation methodology was defined by the US Environment Protection Agency’s (US EPA) methodology [56,57]. The results showed that the estimated daily intake of Li via beverage ingestion (0.0002–0.89 µg/kg_bw_/day) is considerably lower than in the case of food ingestion (0.006–3.76 µg/kg_bw_/day) (Figure 2A,B). The descending order of the average value of EDI_Li_ via food and beverage ingestion decreases as follows: leafy vegetables > fructose solano vegetables > bulbous vegetables > leguminous vegetables > eggs > root vegetables > ciders > fruit juices > beers > meats > red wines > bottled water > white wines > fruits > coffee (Figure 2). The estimated daily intake associated with beverage ingestion, calculated in the present study, is mainly comparable to or lower than other reports. Environmental Li exposure and population diet intake can vary significantly depending on regions, and more or less rely on Li content in food and beverages. Several examples can be referenced for an adult (70 kg body weight) diet, including Italy (0.038 μg/kg_bw_/day) [57]; Belgium (0.12 μg/kg_bw_/day) [98]; France (0.69 μg/kg_bw_/day) [83]; the UK (0.23 μg/kg_bw_/day) [99]; Vietnam (0.51 μg/kg_bw_/day) [100]; New Zealand (0.29–0.41 μg/kg_bw_/day) [101]; and Canary Islands (0.05 mg/kg_bw_/day) [17]. Similar studies confirmed significant daily Li intake via ingesting fresh fruits, vegetables, milk, and dairy products [55,57].

The EPA-US report indicates a provisional reference dose (p-RfD) for the noncancer assessment of daily oral exposure effects for a human lifetime [102]. Based on this reference (p-R_f_D of 2 µg/kg_bw_/day), the estimated daily Li intake was below the provisional subchronic and chronic reference dose for 99.65% of the analyzed samples [102]. The EDI exceeded the provisional subchronic and chronic reference dose in bulbous vegetables (4.87%) and fructose solano vegetables (1.93%) (Figure 2A,B). The maximum values of estimated daily intake are 2.36 and 3.76 µg/kg_bw_/day (bulbous vegetables) and 3.28 µg/kg_bw_/day (fructose solano vegetables), which can raise discussions but not concerns regarding the safety of products for daily consumption. Even so, it must be mentioned that the reference dose does not assume a future human health issue based on higher dose ingestion since it indicates a threshold under which effects will not occur. Statistics regarding food products consumed in Romania and the Li dose measured showed that a higher concentration than 13.47 mg/kg (bulbous vegetables) and 11.33 mg/kg (fructose solano vegetables) would exceed the p-R_f_D reference. Since the R_f_D is expected to evaluate the exposure for various endpoints, after which the effects are correlated with a causal relationship with contaminant concentration, the results do not pose significant concerns regarding toxicological effects in humans. In various regions, reports indicated that humans ingest higher Li doses based on rich environmental concentrations interconnected with the food chain, which were not associated negatively with human health issues each time. Once again, we must state that future studies combining adverse health effects and dietary practices must be conducted on large-scale human collectivities, especially in areas with excessive Li content found in the present study. Although there are multiple studies on the positive relationship between Li content and depressed mood in bipolar affective disorder [103,104,105], no study showed a direct association between Li content in food and human diseases after long-term ingestion.

Li is considered one of the most recent emerging pollutants under concern [106]. The literature showed the provisional recommended intake of 1.0 mg/day Li for a 70 kg adult in dietary intake and the therapeutic oral doses of Li, usually within 600–1200 mg/day [31,64,107]. However, bioaccumulation and trophic transfer via food chains on human health are largely unknown [108]. Li has been used in pharmacology, and it has a phase-prophylactic and antimanic effect in bipolar disorder, being effective as an augmentation to antidepressants, as well as in the prevention of the recurrence of depressive episodes in major depressive disorder. However, long-term Li therapy and exposure via elevated concentration can also cause significant side effects, including thyroid abnormalities, renal tubular damage, and edema. Given the biological effects, the importance of collecting and creating databases with analytical measurements on Li content in foods and beverages is evident. Food consumption can naturally increase Li intake to levels that do not cause adverse side effects. The data of the present study indicated that for most analyzed food and beverage samples, the estimated Li intake dose is below the provisional subchronic and chronic reference dose recommended by EPA-US [102]. Thus, Li levels in tested beverage and food samples could be considered safe for the health of adult consumers. Particular attention must be paid to the constant consumption of several bulbous and fructose solano vegetables.

## 4. Conclusions

The study created a national Romanian-marketed food and beverage database containing Li concentrations for 1071 edible samples to quantify the nutritional benefits of Li, dietary assimilation, and its impact on human health. The results indicated that consuming fruits, milk and dairy products, eggs, and vegetables is a promising source for Li intake. The estimated Li intake via food and beverage ingestion was below the provisional recommended intake level, indicating that consuming the tested beverage and food samples could be considered safe for the population. This dataset could help evaluate the future Li assimilation via food and beverages from Romania. The newly created database can also assess the link between Li content and ecological epidemiology. Li, as a micronutrient, is beneficial for maintaining health and must be consumed regularly in the human diet. Even so, the higher amounts in foods can represent a source of toxicity. Li in trace amounts differs from whole foods; its amount may vary depending on the area of the country or world where the food originates. We evaluated a descending order of the average value of EDI_Li_ via food and beverage ingestion as follows: leafy vegetables > fructose solano vegetables > bulbous vegetables > leguminous vegetables > eggs > root vegetables > ciders > fruit juices > beers > meats > red wines > bottled water > white wines > fruits > coffee. For 99.65% of the analyzed samples, the estimated daily Li intake was below the provisional subchronic and chronic reference doses. The subchronic and chronic reference doses were exceeded in bulbous vegetables (4.87%) and fructose solano vegetables (1.93%) samples, indicating a risk occurrence for possible adverse human health effects.

## Data Availability

The data presented in this study are available on request from the corresponding author.
